# Fully Automatic Sliding Motion Compensated and Simultaneous 4D-CBCT *via* Bilateral Filtering

**DOI:** 10.3389/fonc.2020.568627

**Published:** 2021-01-18

**Authors:** Jun Dang, Tao You, Wenzheng Sun, Hanguan Xiao, Longhao Li, Xiaopin Chen, Chunhua Dai, Ying Li, Yanbo Song, Tao Zhang, Deyu Chen

**Affiliations:** ^1^Department of Oncology, The First Affiliated Hospital of Chongqing Medical University, Chongqing, China; ^2^Department of Radiation Oncology, The Affiliated Hospital of Jiangsu University, Zhenjiang, China; ^3^Department of Radiation Oncology, The Second Affiliated Hospital of Zhejiang University School of Medicine, Hangzhou, China; ^4^School of Artificial Intelligence, Chongqing University of Technology, Chongqing, China

**Keywords:** sliding motion, bilateral filtering, 4D-CBCT, deformable vector fields, simultaneous reconstruction

## Abstract

**Purpose:**

To incorporate the bilateral filtering into the Deformable Vector Field (DVF) based 4D-CBCT reconstruction for realizing a fully automatic sliding motion compensated 4D-CBCT.

**Materials and Methods:**

Initially, a motion compensated simultaneous algebraic reconstruction technique (mSART) is used to generate a high quality reference phase (e.g. 0% phase) by using all phase projections together with the initial 4D-DVFs. The initial 4D-DVF were generated *via* Demons registration between 0% phase and each other phase image. The 4D-DVF will then kept updating by matching the forward projection of the deformed high quality 0% phase with the measured projection of the target phase. The loss function during this optimization contains an projection intensity difference matching criterion plus a DVF smoothing constrain term. We introduce a bilateral filtering kernel into the DVF constrain term to estimate the sliding motion automatically. The bilateral filtering kernel contains three sub-kernels: 1) an spatial domain Guassian kernel; 2) an image intensity domain Guassian kernel; and 3) a DVF domain Guassian kernel. By choosing suitable kernel variances, the sliding motion can be extracted. A non-linear conjugate gradient optimizer was used. We validated the algorithm on a non-uniform rotational B-spline based cardiac-torso (NCAT) phantom and four anonymous patient data. For quantification, we used: 1) the Root-Mean-Square-Error (RMSE) together with the Maximum-Error (MaxE); 2) the Dice coefficient of the extracted lung contour from the final reconstructed images and 3) the relative reconstruction error (RE) to evaluate the algorithm's performance.

**Results:**

For NCAT phantom, the motion trajectory's RMSE/MaxE are 0.796/1.02 mm for bilateral filtering reconstruction; and 2.704/4.08 mm for original reconstruction. For patient pilot study, the 4D-Dice coefficient obtained with bilateral filtering are consistently higher than that without bilateral filtering. Meantime several image content such as the rib position, the heart edge definition, the fibrous structures all has been better corrected with bilateral filtering.

**Conclusion:**

We developed a bilateral filtering based fully automatic sliding motion compensated 4D-CBCT scheme. Both digital phantom and initial patient pilot studies confirmed the improved motion estimation and image reconstruction ability. It can be used as a 4D-CBCT image guidance tool for lung SBRT treatment.

## Introduction

In image-guided radiation therapy (IGRT), 3D Cone Beam CT (CBCT) is extensively applied to check patient positioning before a radiation beam is delivered. However 3D-CBCT is not capable to capture a dynamic moving target and reflect the respiration motion during radiation therapy. Nowadays SBRT (Stereotactic Body Radiotherapy) has been applied widely for lung cancer treatment due to its better treatment effect compared with conventional IMRT (Intensity Modulated Radiation Therapy). At the SBRT treatment stage for lung cancer cases, the patient usually will be performed with a 3D-CBCT to check positioning before SBRT beam on. However in this process, the physician cannot check again if the patient respiration matches with that of the 4D-CT, especially for the GTV region. To compensate for the deficiency, 4D-CBCT was proposed for accurate on board motion tracking. There are different categories of existed 4D-CBCT reconstruction schemes. The first category is employed to increase the acquired projection number under each gantry angle by performing multiple gantry rotation or reducing the gantry rotation speed (1, 2). But it prolongs the imaging time and increases the imaging dose. The second 4D-CBCT category is the non-local mean/Total Variation (TV)-based algorithms (3–5). The TV method supplies a qualified noise suppressed image but it over-smoothed tiny structures and further deteriorate the image quality of the low contrast region. The third category is the full data initialization-based reconstruction such as the McKinnon-Bates (MKB) algorithm (6, 7) and the prior image constrained compressed sensing (PICCS)-based algorithm (7, 8). However, the residual motion will transmit artifacts from the initial reconstruction to the final images. And the fourth category is the low-rank models (9) and the framelet (5, 10) based reconstruction. However, the low rank method cannot fully realize the time differentiation, and both of these two methods are lack of clinical supporting results feasibility check. In recent years, the Deformable Vector Field (DVF)-based 4D-CBCT image reconstruction algorithm has shown an advantage for high-quality 4D-CBCT reconstruction (11–14). However, most of those methods assume the lung moves along an uniform path and ignored the lung's non-average local motion (e.g. sliding motion). This assumption is not true since sliding motion exists widely at the interfaces between moving organs such as the lung and the chest wall's interface. A few studies have tried to model the sliding motion via lung boundary segmentation (12). But its clinical translation is hindered due to its ineluctable requirement of lung boundary half automatic segmentation.

In this study, we develop a fully automatic sliding motion compensated 4D-CBCT reconstruction algorithm in a fundamentally different way by using bilateral filtering. This algorithm performs bilateral filtering on the DVF during the motion optimization process. Bilateral filtering has been previously utilized for estimating sliding motion for 4D-CT (15). But here we adapt this technique to 4D-CBCT, which is a more challenging scenario. Accurate 4D-DVF estimation from 4D-CBCT imaging geometry, especially for sliding motion extraction, is more difficult than that of 4D-CT. This is not only because the acquired CBCT projections are contaminated with serious scatters but also because the available projection number per phase are quite limited due to conventional 1 min clinical scanning protocol. We estimate the 4D-DVF by matching the measured projection of each target phase with the deformed phase 0%'s Digital Reconstructed Radiography (DRR). Meantime we incorporate bilateral filtering into the 4D-DVF estimation process for sliding motion modeling. A non-linear conjugate gradient optimizer is used for this optimization process.

Our results indicate that the bilateral filtering-based motion modeling and reconstruction is capable of better sliding motion modeling. For algorithm validation, we applied a non-uniform rotational B-spline that is based on a cardiac-torso (NCAT) phantom. Subsequently, four patient data with IRB approval were used to perform an initial pilot clinical validation.

## Methods and Materials

### The Bilateral Filtering Based Simultaneous 4D-CBCT Image Reconstruction Algorithm

We first make a short review of the original simultaneous motion compensated reconstruction algorithm. There are two steps in the algorithm: 1) reconstruct a high quality phase 0% image using all acquired projections with motion compensated SART (Simultaneous Algebraic Reconstruction Technique, mSART). The motion compensated SART is mathematically described in equation (1). Then step 2): estimate the 4D-DVF by matching each phase's measured projection with the DRR (Digitally Reconstructed Radiography) of the deformed phase 0%. These two steps are performed in an interleaved fashion to allow a converged energy function curve. The loss function was designed into a symmetrical form to ensure an inverse consistent DVF solution, see equation (2). Once the 4D-DVF optimized solution were obtained it will be used to deform the final iterative reconstructed high quality phase 0% image to get the final high quality 4D-CBCT[see equation (6)]. Mathematically, the above mentioned steps can be expressed as follow:

Let $p^{t}=\left(p_{1}^{t},p_{2}^{t},\ldots p_{I}^{t}\right)$ denote the log-transformed 4D-CBCT projection (i.e., line integral) from phase t, and $\mu^{t}=\left(\mu_{1}^{t},\mu_{2}^{t},\ldots \mu_{J}^{t}\right)$ denote the attenuation coefficients of phase t image, the modified mSART is given by (1):

(1) $$\;\mu_{j}^{0,(k+1)}=\mu_{j}^{0,(k)}+\frac{\Sigma_{\text{jn}}d_{\text{jn}}^{t\to 0}\Sigma_{i}\left[a_{\text{in}}\frac{p_{1}^{t}-\Sigma_{n}a_{\text{in}}\mu_{n}^{t,(k)}}{\Sigma_{n=1}^{J}a_{\text{in}}}\right]}{\Sigma_{i}a_{\text{in}}}$$

where k is the iteration step, j is the voxel index of phase 0%, n is the voxel index of phase t. a_in_ is the intersection length of projection ray i with voxel n, which is obtained by a ray-tracing technique (16). $d_{\text{jn}}^{t\to 0}$ denotes the element of the inverse DVF that deforms phase t to phase 0. The initial image $\mu_{j}^{0,(0)}$ is first reconstructed by the TV minimization (17) reconstruction to achieve a noise suppressed initial 0% phase 0%. For projection matching, an inverse consistent DVF estimation is applied by designing a symmetric energy function:

$$f_{1}(v^{0\to t})={\left\|\text{pt}-A\mu^{0}\left(x+v^{0\to t}\right)\right\|}_{l_{2}}^{2}+\beta \varphi \left(v^{0\to t}\right)$$

$$f_{2}(v^{t\to 0})={\left\|p^{0}-A\mu^{t}\left(x+v^{t\to 0}\right)\right\|}_{l_{2}}^{2}+\beta \varphi \left(v^{t\to 0}\right)$$

(2) $$\text{s.t. }v^{0\to t}\left(x+v^{t\to 0}\right)+v^{t\to 0}=v^{t\to 0}\left(x+v^{0\to t}\right)+v^{0\to t}=0$$

f_1_ and f_2_ denote the symmetric energy function. 0 stands for phase 0%, t stands for any other phase t. **A** is the projection matrix. x stands for the voxel of image μ^0^ or μ^t^.v^0→t^ denotes the forward DVF element for each voxel, and v^t→0^ denotes the inverse DVF element for each voxel. ${\left\|\text{pt}-A\mu^{0}\left(x+v^{0\to t}\right)\right\|}_{l_{2}}^{2}$ and ${\left\|p^{0}-A\mu^{t}\left(x+v^{t\to 0}\right)\right\|}_{l_{2}}^{2}$ are the data fidelity terms of the inverse consistent loss function. φ(v^0→t^) and φ(v^t→0^) are the corresponding regularization terms. β controls the trade-off between the data fidelity term and smoothing regularization term φ(v). If the lung is supposed to have an isotropic motion mode, φ(v) will be designed by:

(3) $$\varphi \left(v\right)=\sum_{v\in R^{3}}\sum_{i=1}^{3}\sum_{j=1}^{3}{\left(\frac{\partial {\text{v}}_{\text{i}}}{\partial {\text{x}}_{\text{j}}}\right)}^{2}$$

where (∂v_i_)/(∂x_j_) denotes the difference between neighboring voxels for each DVF component along three directions. Index “i” stands for the DVF component along x, y, and z direction. Index “j” stands for one of three Cartesian coordinates; “v_i_” stands for the DVF element along each Cartesian coordinate direction; “x_j_” stands for each image voxel along each Cartesian coordinate direction.

We take sliding motion into account and re-designed the bilateral filtering based regularization term:

(4) $$\varphi \left(v\right)=\sum_{v\in R^{3}}\sum_{i=1}^{3}\sum_{j=1,y_{k=1,\ldots N}\in N\left({\text{x}}_{\text{j}}\right)}^{3}\left({\text{G}}_{\text{x}}\left({\text{x}}_{\text{j}},{\text{y}}_{\text{k}},\sigma_{x}^{2}\right)\cdot G_{\mu }\left(\mu^{t}\left({\text{x}}_{\text{j}}\right),\mu^{t}\left({\text{y}}_{\text{k}}\right),\sigma_{\mu }^{2}\right)\cdot G_{{\text{v}}_{\text{i}}}\left({\text{v}}_{\text{i}}\left({\text{x}}_{\text{j}}\right),{\text{v}}_{\text{i}}\left({\text{y}}_{\text{k}}\right),\sigma_{v}^{2}\right){\left(\frac{\partial {\text{v}}_{\text{i}}}{\partial {\text{x}}_{\text{j}}}\right)}^{2}\right)$$

with

$${\text{G}}_{\text{x}}\left({\text{x}}_{\text{j}},{\text{y}}_{\text{k}},\sigma_{x}^{2}\right)=\exp\left(-\frac{{\left({\text{x}}_{\text{j}}-{\text{y}}_{\text{k}}\right)}^{T}\left({\text{x}}_{\text{j}}-{\text{y}}_{\text{k}}\right)}{2\sigma_{x}^{2}}\right)$$

$$G_{\mu }\left(\mu^{t}\left({\text{x}}_{\text{j}}\right),\mu^{t}\left({\text{y}}_{\text{k}}\right),\sigma_{\mu }^{2}\right)=\exp\left(-\frac{{\left\|\mu^{t}\left({\text{x}}_{\text{j}}\right)-\mu^{t}\left({\text{y}}_{\text{k}}\right)\right\|}^{2}}{2\sigma_{\mu }^{2}}\right)$$

$$G_{{\text{v}}_{\text{i}}}\left({\text{v}}_{\text{i}}\left({\text{x}}_{\text{j}}\right),{\text{v}}_{\text{i}}\left({\text{y}}_{\text{k}}\right),\sigma_{v}^{2}\right)=\exp\left(-\frac{{\left({\text{v}}_{\text{i}}\left({\text{x}}_{\text{j}}\right)-{\text{v}}_{\text{i}}\left({\text{y}}_{\text{k}}\right)\right)}^{T}\left({\text{v}}_{\text{i}}\left({\text{x}}_{\text{j}}\right)-{\text{v}}_{\text{i}}\left({\text{y}}_{\text{k}}\right)\right)}{2\sigma_{v}^{2}}\right)$$

G_x_ is the Gaussian kernel on the spatial domain with the variance $\sigma_{x}^{2}$; G_μ_ is another image domain-based Gaussian kernel with the variance $\sigma_{\mu }^{2}$; and G_v_ is the DVF domain Gaussian kernel with the variance $\sigma_{x}^{2}$. The index “i”, “j”, “v_i_”, and “x_j_” have the same meaning that mentioned in equation (3). Meantime “x_j_” is also the central voxel in each bilateral filter kernel. “y_j_” represents the neighborhood voxel surround x_j_, with a max number of N. k is the surround voxel index of “y_j_”. For implementation, the gradient ∇φ(v)|_v_ is calculated within the 3x3x3 neighborhood that surrounds each voxel of interest. A nonlinear conjugate gradient optimizer was used to estimate the final DVF solution. We also give the gradient of φ(v):

(5) $${\nabla \varphi \left(v\right)|}_{v}=\sum_{v\in R^{3}}\sum_{i=1}^{3}\sum_{j=1,y_{k=1,\ldots ,N}\in N\left({\text{x}}_{\text{j}}\right)}^{3}\left({\text{G}}_{\text{x}}\left({\text{x}}_{\text{j}},{\text{y}}_{\text{k}},\sigma_{x}^{2}\right)\cdot G_{\mu }\left(\mu^{t}\left({\text{x}}_{\text{j}}\right),\mu^{t}\left({\text{y}}_{\text{k}}\right),\sigma_{\mu }^{2}\right)\cdot {\text{G}}_{\text{v}}\left({\text{v}}_{\text{i}}\left({\text{x}}_{\text{j}}\right),v_{i}\left({\text{y}}_{\text{k}}\right),\sigma_{v}^{2}\right)\left({\text{v}}_{\text{i}}\left({\text{x}}_{\text{j}}\right),v_{i}\left({\text{y}}_{\text{k}}\right)\right)\right)$$

When high quality phase 0% $\mu_{j}^{2,(k)}$ is finally obtained, each other 4D phase image $\mu_{n}^{t,(k)}$ can be obtained by deforming $\mu_{j}^{0,(k)}$ with the final optimized 4D-DVFs. See equation below:

(6) $$\mu_{n}^{t,(k)}=\sum_{j}d_{\text{jn}}^{0\to t}\mu_{j}^{0,(k)}$$

To accelerate the energy function's convergence, we need to generate the initial 4D-DVF to start the optimization process. The measured CBCT projections are initially sorted into 4D for an initial 4D-CBCT TV reconstruction (3). A Demons registration algorithm (18) was then employed to obtain the 4D-DVF initials between each phase and the 0% phase.

The pseudo code of the algorithm is given as follows:

1. initial input data preparation: TV image reconstruction (3) for phase 0% to 90%; use the TV images generated in (a) to generate the initial 4D-DVFs between phase 0% and each other phase via Demons registration
2. projection domain registration for 4D-DVF optimization: use all measured CBCT projections and the initial 4D-DVF to generate the initial phase 0% motion compensated image reconstruction via mSART algorithm, see eq. (1) register the measured CBCT projection with the forward projection (e.g., DRR) of the deformed high-quality phase 0% image obtained in (c) generate the image domain DVF for each phase via nonlinear conjugate gradient optimizer.

The loss function curve was draw with the DVF optimization iteration. And the optimization stops if the curve converges. For calculation acceleration, the code is run on a GPU card (Geforce GTX 980, NVIDA, Santa Clara, CA) for parallel computation. The data processing time will be discussed in the discussion part.

**Figure 1:**
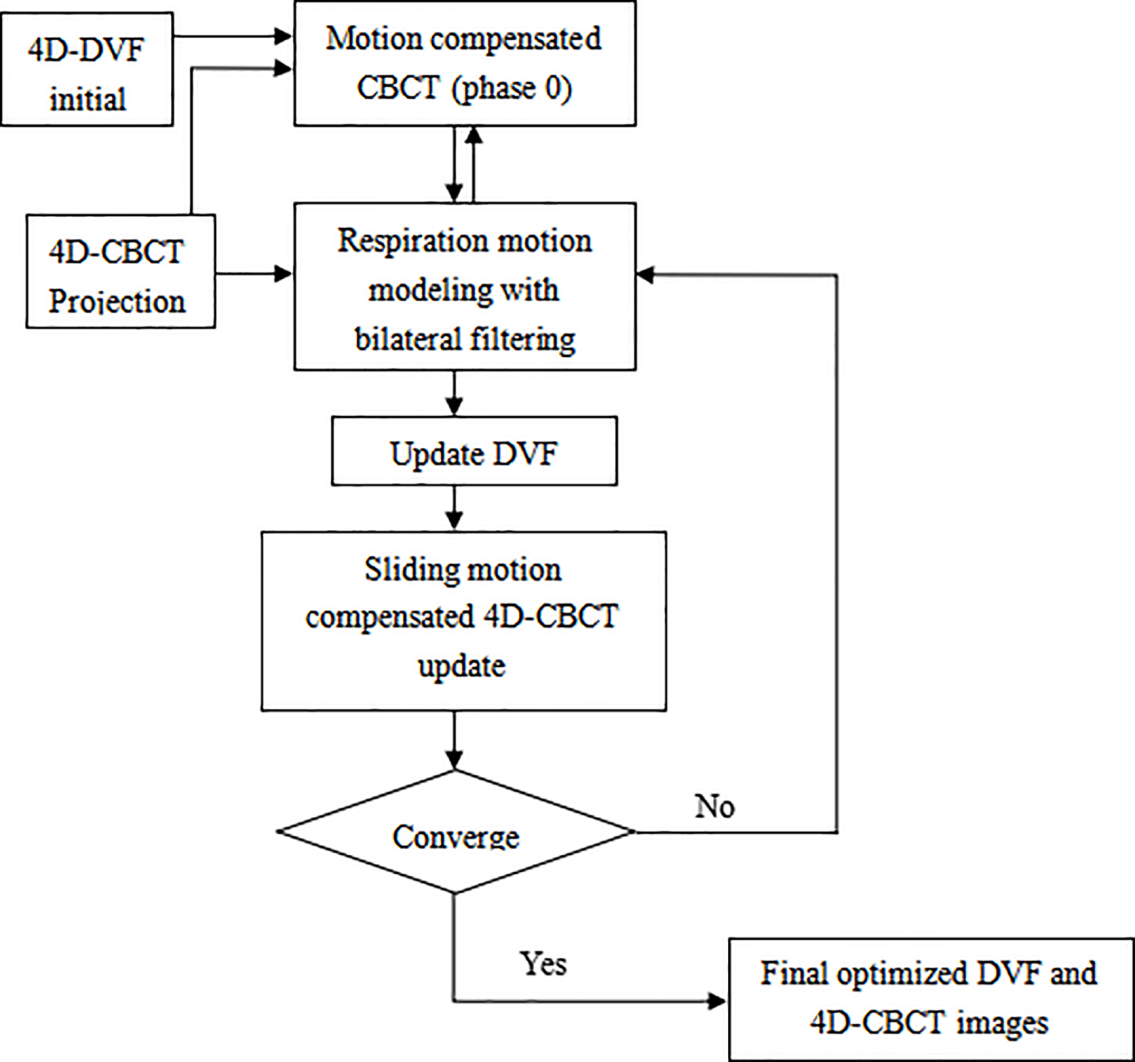
Overflow chart of the bilateral filtering based sliding motion compensated 4D-CBCT reconstruction scheme.

The algorithm work flow chart is given below (**Figure 1**):

### Algorithm Validation Experiments Design

#### The Digital NCAT Phantom Experiment

The NCAT phantom was first used to evaluate the performance of the bilateral filtering based sliding motion estimation algorithm. 10 breathing phase of 4D NCAT were simulated with respiration period of 4 s. The maximum diaphragm motion along Superior-Interior (SI) is 20 mm and the maximum chest Anterior-Posterior (AP) motion is 12 mm. The projections of 10 phases with 20 views per phase were used for the DVF estimation and 4D-CBCT reconstruction. The phantom image size is 256 x 256 x 150 with a voxel size of 1x1x1 mm^3^. The projection size is 300 x 240 x 20 view per phase with projection voxel size of 1x1 mm^2^. We compared the bilateral filtering reconstruction results with the lung segmentation based (12) algorithm, the original simultaneous reconstruction algorithm (11) and the ground truth reference for quantitative evaluation. For motion tracking comparison, the 4D NCAT motion trajectory along z-direction are extracted from the heart edge in the coronal view slice for quantitative evaluation.

#### Pilot Patient Data Evaluation

Four sets of lung cancer patient data were used to perform an initial pilot clinical validation of the bilateral filtering-based 4D-CBCT reconstruction algorithm. Using an IRB approved protocol (MD Anderson with IRB# 00-202), the patients were scanned in full fan mode for 4–6 min to acquire approximately 2000 projections. The acquired projections were then sorted by phase binning into 10 phases. In this manner, the number of average projections per phase was approximately 200, and TV minimization reconstruction was applied to reconstruct the high-quality 4D-CBCT that can serve as the patient reference for clinical results quantification. To simulate an approximate 1-min CBCT data we down-sampled the acquired 4D full projections until the average projection number per phase decreased to ~40. We performed the original simultaneous reconstruction and the bilateral filtering-based sliding constrained reconstruction for quantitative comparisons with the ground truth. Here we need to clarify that although the down-sampling helps to mimic a 1-min CBCT, one still cannot getting a real 1-min CBCT data. Even with the same number of projection per phase, 6-min CBCT scan is still better than 1-min case because the projections are further spread out in 6-min case. Down-sampling only helps to mimic an approximate 1-min CBCT case for algorithm testing.

### Selection of σ_x_, σ_μ_, and σ_v_

The selection criteria of **σ**_x_, **σ**_μ_, and **σ**_v_ will dramatically influence the final DVF solution. It's relatively easy to determine **σ**_x_, **σ**_μ_. Excessively large or small **σ**_x_ (spatial smoothness) will either over-smooth the image content or prevent it from sufficiently capturing the local sparse features. The voxel size is 1 mm^3^ for the NCAT phantom data and 2 mm^3^ for the patient data. Hence the reasonable spatial variance **σ**_x_ should not be smaller than 2 mm. With several round testing, we determined **σ**_x_ = 3 mm gives the best results for the reconstructed images both for the NCAT phantom and the patient data. The image will be over-smoothed if **σ**_x_ is larger than 3 mm. **σ**_μ_ controls the image intensity domain smoothness between the interface of the lung part and the chest wall. We set it equal to the difference between the lung tissue and the surrounding chest cavity tissue to retain the nature intensity transit from the chest cavity boundary to the lung inner part. For the NCAT phantom data, **σ**_μ_ = 0.03 mm^-1^ gives the best results, and for the patient pilot data, **σ**_μ_ = 0.02 mm^-1^ gives the best results. The most difficult part is to determine **σ**_v_ for extracting the sliding motion. Theoretically, **σ**_v_ should be larger than the DVF difference between two points at a distance smaller than **σ**_x_. However at the pleural cavity region **σ**_v_ should be smaller than the DVF intensity difference (15) between the two points. To avoid motion over-segmentation, we set that only sharp discontinuities (e.g., large sliding motion) can be captured. In our former work (12), we compared the results obtained from the original simultaneous reconstruction method and the ground truth and discovered that the sliding motion estimation error at the heart edge site is approximately 7.5 mm. Hence, we suggest that 10 mm is a relatively large amount of sliding motion. With this assumption, we tested several **σ**_v_ values and determined that **σ**_v_ = 2.5 mm gives reasonable results for NCAT data.

For the patient pilot study, we also compared the original simultaneous reconstruction results with the high-quality patient reference. We discovered that the rib position has a maximum motion error of approximately 5~6 mm at the pleural cavity site. Hence, we suggest that 6 mm is a relatively large sliding motion amount. With this assumption, we establish 2 mm of **σ**_v_ for the patient pilot test and obtain the desired results.

### Evaluation Criteria

#### Tumor Motion Accuracy

The tumor motion trajectory was extracted from the reconstructed images and the ground truth. The root mean square error (RMSE) of the estimated tumor position is analyzed to quantify motion estimation accuracy with sliding motion constraint.

(7) $$\text{RMSE}=\sqrt{\frac{1}{3}\times \sum_{\text{ph}=1}^{9}{\left(Pos_{ph}^{R}-Pos_{ph}^{T}\right)}^{2}}$$

where ${Pos}_{ph}^{R}$ denotes the estimated image feature point position for the *ph^th^* phase and ${Pos}_{ph}^{T}$ denotes the corresponding position from the ground truth. MaxE is defined as the maximum error of the tumor position extracted from all 9 phases.

#### Dice Coefficient

After the final 4D reconstruction is finished, we used the dice coefficient to measure the segmented lung boundary contours to see whether sliding motion compensated result have more contour similarity compared with the truth reference. The segmentation is performed via ITK snap software tool. Let A be the contour area obtained from result with or without sliding motion compensation, and B is the contour from the truth reference. The dice coefficient s given by:

(8) $$S=\frac{2\left|A\cap B\right|}{\left|A\right|+\left|B\right|}$$

In our study, we use the voxel number within the organ contour as a surrogate of the exact area.

#### Relative Reconstruction Error

The relative error (RE) between the reconstructed 4D-CBCT with sliding modeling and the ground truth/reference was used to quantify the image reconstruction accuracy by defining

(9) $$RE=\sqrt{\frac{\sum {\left(u_{R}\left(x\right)-\mu_{T}\left(x\right)\right)}^{2}}{\sum {\left(u_{T}\left(x\right)\right)}^{2}}}\times 100\%$$

where *u_T_*(*x*) stands for the phantom ground truth, *u_R_*(*x*) is the reconstructed image.

#### Parameter Sensitivity σ_v_ for NCAT Phantom Experiment

Since the bilateral filtering kernels have multiple parameters (e.g., σ_x_, σ_μ_, and σ_v_), a sensitivity analysis is necessary to clarify how these parameters influence the 4D-DVF estimation. The spatial domain parameter σ_x_ and the voxel intensity domain parameter σ_μ_'s selection criteria are simple and clearly decided. However, the most challenging parameter is σ_v_. We performed a NCAT phantom test of the 4D-CBCT reconstruction algorithm with different σ_v_ values, which range from 1.0 to 5.0 per 0.5 step increase. The σ_x_ was set to 3 mm, and σ_μ_ was set to 0.03 mm^-1^. Since digital phantom data already eliminate contamination resources such as scattering and noise, the obtained reconstruction error is mainly caused by σ_v_.

## Results

### NCAT Phantom Results

**Figure 2:**
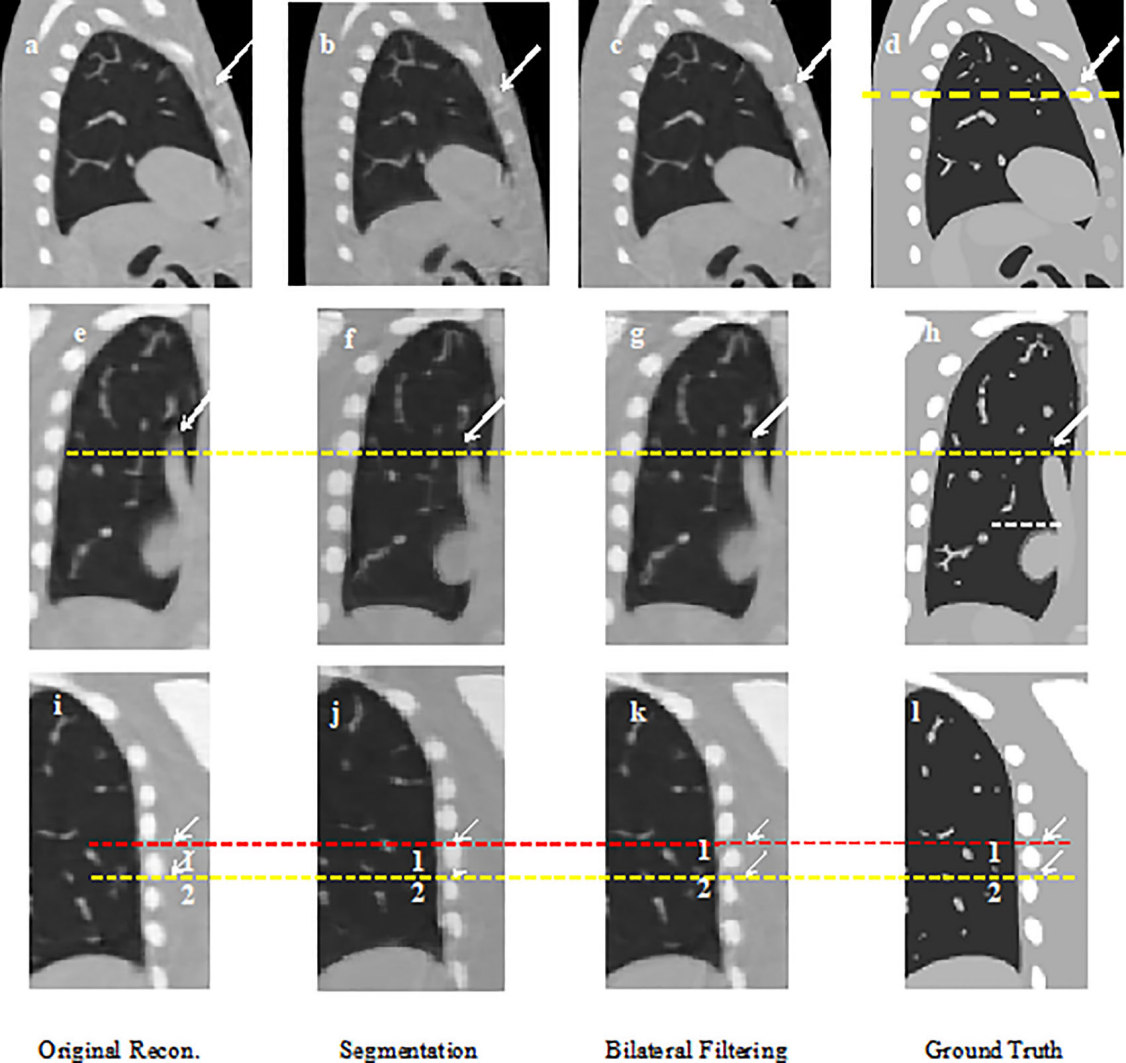
NCAT phantom results comparison: **(A)** Sagittal view without sliding motion modeling; **(B)** Sagittal view with segmentation based motion modeling; **(C)** Sagittal view with bilateral filtering motion modeling **(D)** Sagittal view ground truth; **(E)** Coronal view without sliding motion modeling; **(F)** Coronal view with segmentation based motion modeling; **(G)** Coronal view with bilateral filtering motion modeling; **(H)** Coronal view ground truth; **(I)** rib position without sliding motion modeling; **(J)** rib position with segmentation based motion modeling; **(K)** rib position with bilateral filtering motion modeling; **(L)** rib position ground truth.

**Figure 2** shows the 40% phase reconstructed images obtained from the original reconstruction (e.g. without sliding motion modeling), the segmentation based reconstruction, and the bilateral filtering based reconstruction. **Figure 2A** shows the sagittal view of the reconstructed 40% phase obtained from the original reconstruction; **Figure 2B** shows the same sagittal slice reconstructed from the segmentation based reconstruction; **Figure 2C** shows the sagittal slice reconstructed from the bilateral filtering reconstruction; **Figure 2D** shows the phantom ground truth; the white arrow labels the rib, which can be seen clearly in the bilateral filtering based reconstructed image and the ground truth. The rib is also partially visible in the segmentation based reconstructed image. But it is hardly visible in the original reconstructed image (e.g. without sliding motion modeling). The white arrows clearly labeled the rib comparison. **Figures 2E–H** are the regions of interest (ROI), where sliding motion exists at the heart edge and the vein site. The vein (indicated by a yellow dotted line) is more accurately reconstructed (e.g., vein length has been better reconstructed, see the white arrows) with bilateral filtering and the segmentation based reconstruction. **Figures 2I–L** show the rib position reconstruction differences between the original reconstruction, the segmentation based reconstruction, the bilateral filtering reconstruction and the ground truth. In **Figures 2J, K**, rib top edges 1 and 2 match the ground truth with **Figure 2L** compared with that of the original reconstruction in **Figure 2I**.

#### NCAT Phantom Motion Trajectory Result

**Figure 3:**
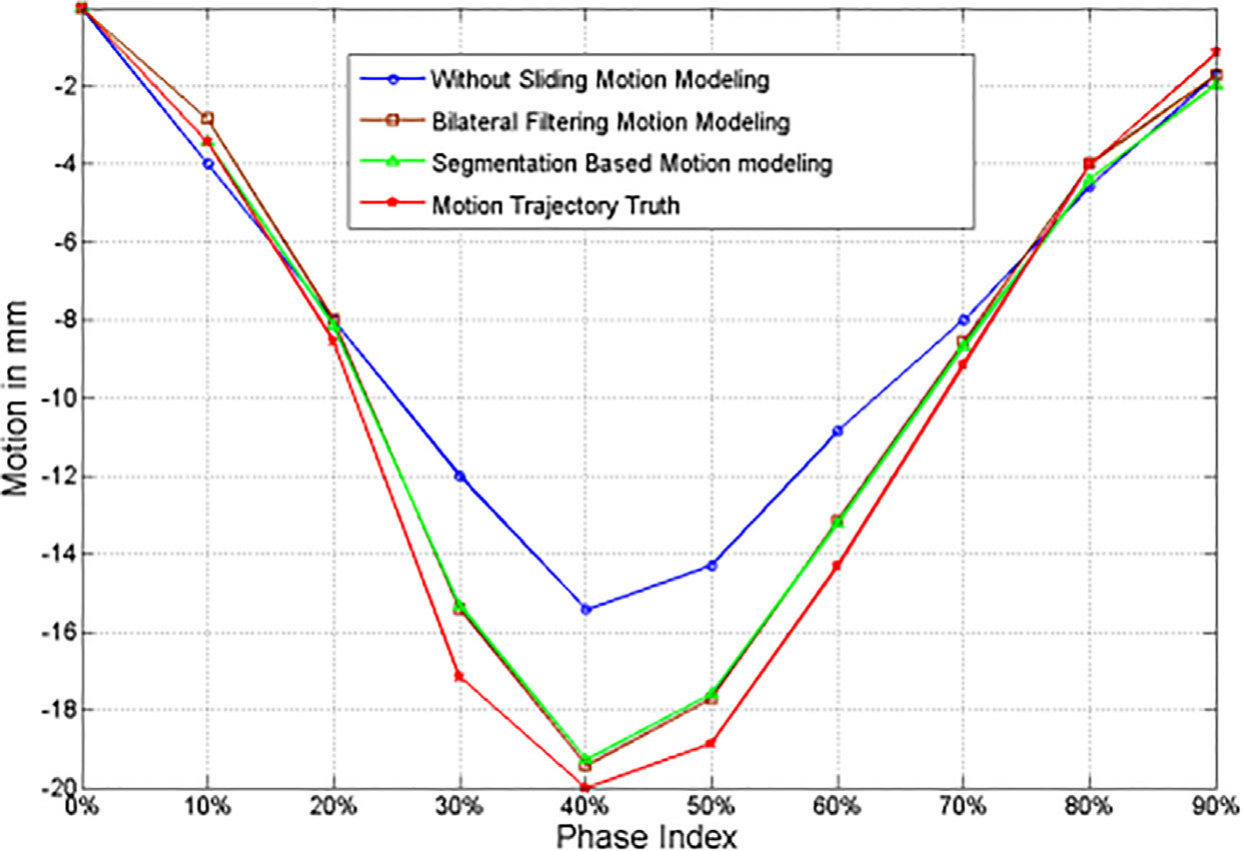
z-axis heart motion trajectories extracted from the NCAT phantom ROI truth and the corresponding ROI images from the original simultaneous reconstruction (e.g. without bilateral filtering), the segmentation based sliding motion estimation, and the bilateral filtering based sliding motion estimation.

The 4D NCAT motion trajectory along the z-direction are extracted from the heart edge in the coronal view slice [refer to the dotted line position in **Figure 2F**]. The dotted line position is detected from a ROI binary image by establishing a uniform threshold for each phase. The detected dotted line positions are used to plot the motion trajectory. **Figure 3** shows the 4D motion trajectory extracted from the original reconstruction without sliding motion modeling, the segmentation based sliding motion modeling, the bilateral filtering based sliding motion modeling, and the motion ground truth. The figure indicates that the trajectory extracted both with bilateral filtering and segmentation-based sliding motion modeling matches better with the ground truth. We consider each of the trajectory's motion amplitude for the RMSE calculation and determine that the trajectory's RMSE and MaxE are 0.796 mm and 1.02 mm for the bilateral filtering-based results. Meantime the segmentation based RMSE/MaxE are quite close to that of the bilateral filtering based results. The original reconstruction result’s RMSE and MaxE are 2.704 mm and 4.08 mm, respectively.

#### Dice Coefficient

We extract the 4D lung boundaries (by ITK-SNAP software) from the original simultaneous reconstruction, the segmentation based and the bilateral filtering reconstruction. The 4D Dice coefficients extracted from the NCAT phantom experiment with each different motion modeling scheme are summarized in **Table 1**. Both of the segmentation based and the bilateral filtering-based Dice coefficients are consistently larger than that from the original simultaneous reconstruction. The results indicate that the lung boundary can be more accurately segmented with segmentation based and bilateral filtering based motion estimation compared with that of the original reconstruction.

**Table 1:** 4D Dice coefficients between NCAT phantom results obtained from the original simultaneous reconstruction vs. the segmentation based reconstruction and the bilateral filtering reconstruction.

Phase	0%	10%	20%	30%	40%	50%	60%	70%	80%	90%	Avg. Dice
Original Recon.	0.989	0.980	0.975	0.960	0.930	0.928	0.925	0.965	0.970	0.975	0.960
Segmentation Recon.	0.998	0.998	0.991	0.990	0.989	0.986	0.979	0.986	0.988	0.986	0.990
Bilateral filtering Recon	0.998	0.998	0.992	0.989	0.985	0.985	0.980	0.988	0.992	0.985	0.990

#### Parameter Sensitivity σ_v_ for NCAT Phantom Experiment Analysis

**Figure 4:**
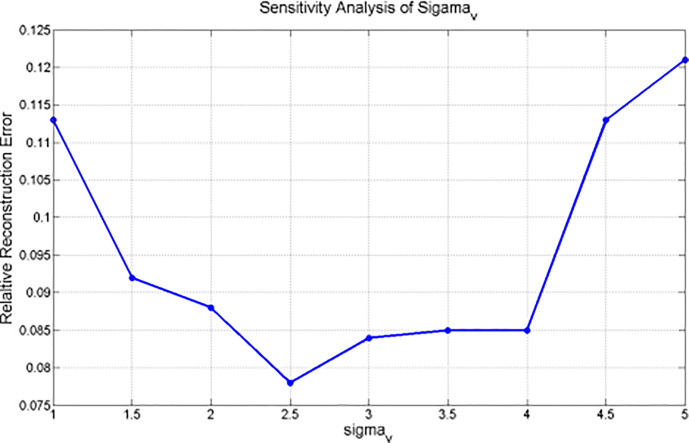
Sensitivity analysis of σ_v_ for NCAT phantom based experiment.

The correspondence between the relative reconstruction error and σ_v_ is plotted in **Figure 4**. This figure indicates that the minimum relative reconstruction error can be obtained with σ_v_ = 2.5 mm.

#### Profiles for NCAT Result

**Figure 5:**
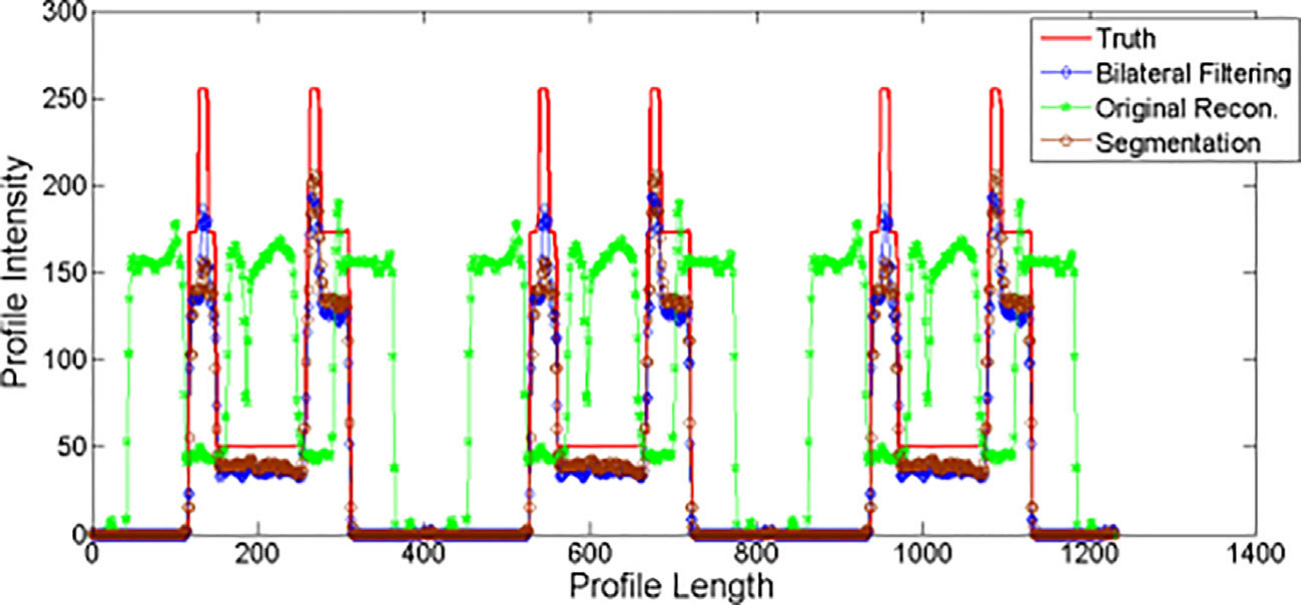
NCAT phantom experiment profiles comparison.

We plot the profiles for the NCAT phantom result in **Figure 5**. The profile is plotted by the yellow dot line in **Figure 2C**. Red line stands for the phantom profile reference (e.g. Truth); blue line stands for the bilateral filtering based profile, brown line stands for the profile obtained from the segmentation based reconstruction, and the green line stands for the original reconstruction based profile. The sharp peak in red line stands for the rib that the dot line comes across. The profile shows that the blue line keeps the same correspondence with the red line while the green line totally missed the rib.

### Patient Pilot Study

**Figure 6:**
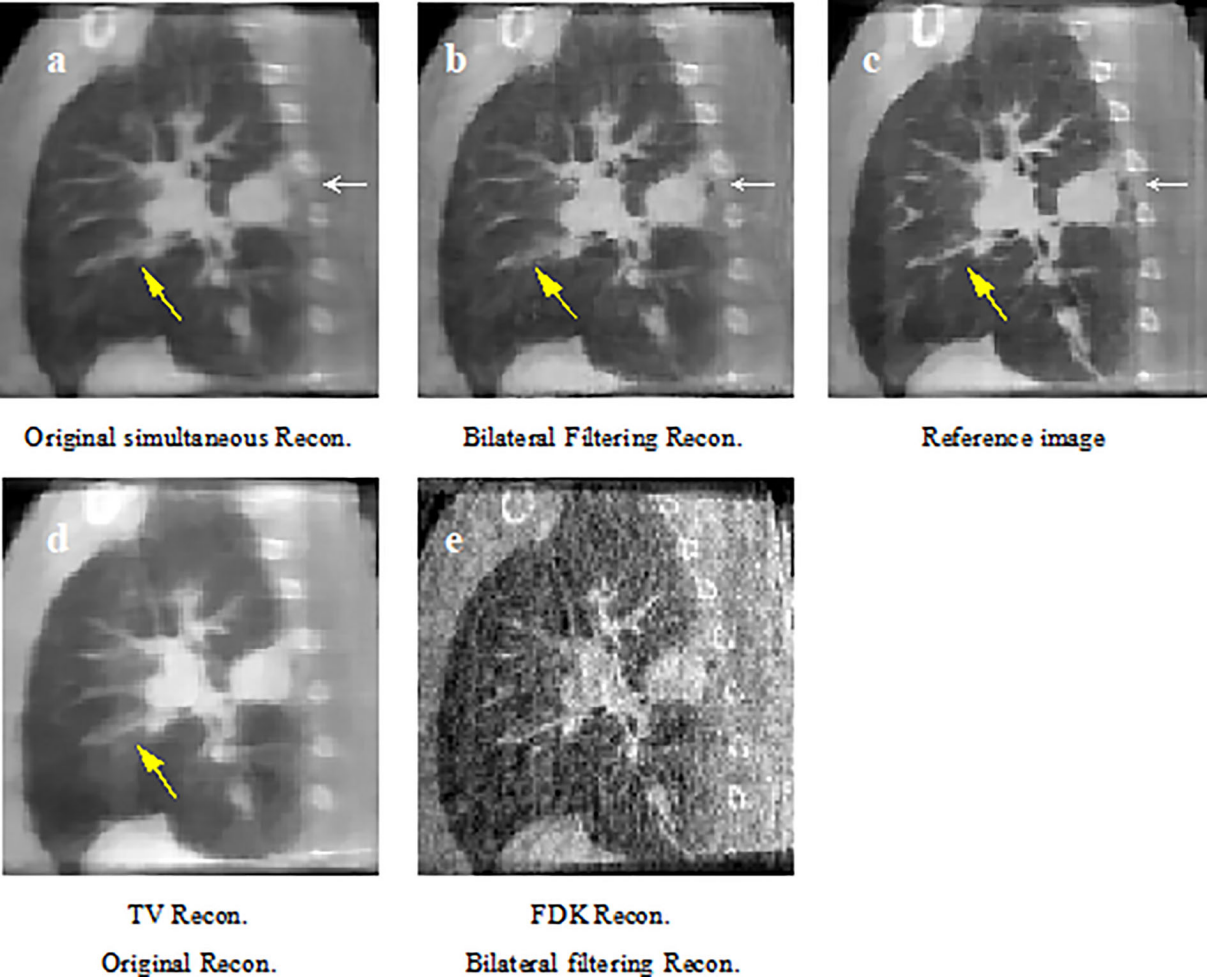
Patient 1 reconstruction results comparison. **(A)** reconstructed sagittal view of the original simultaneous reconstruction; **(B)** reconstructed sagittal view with bilateral filtering; **(C)** patient 1 sagittal view reference; **(D)** corresponding sagittal view via TV reconstruction; **(E)** corresponding sagittal view *via* FDK reconstruction.

**Figure 7:**
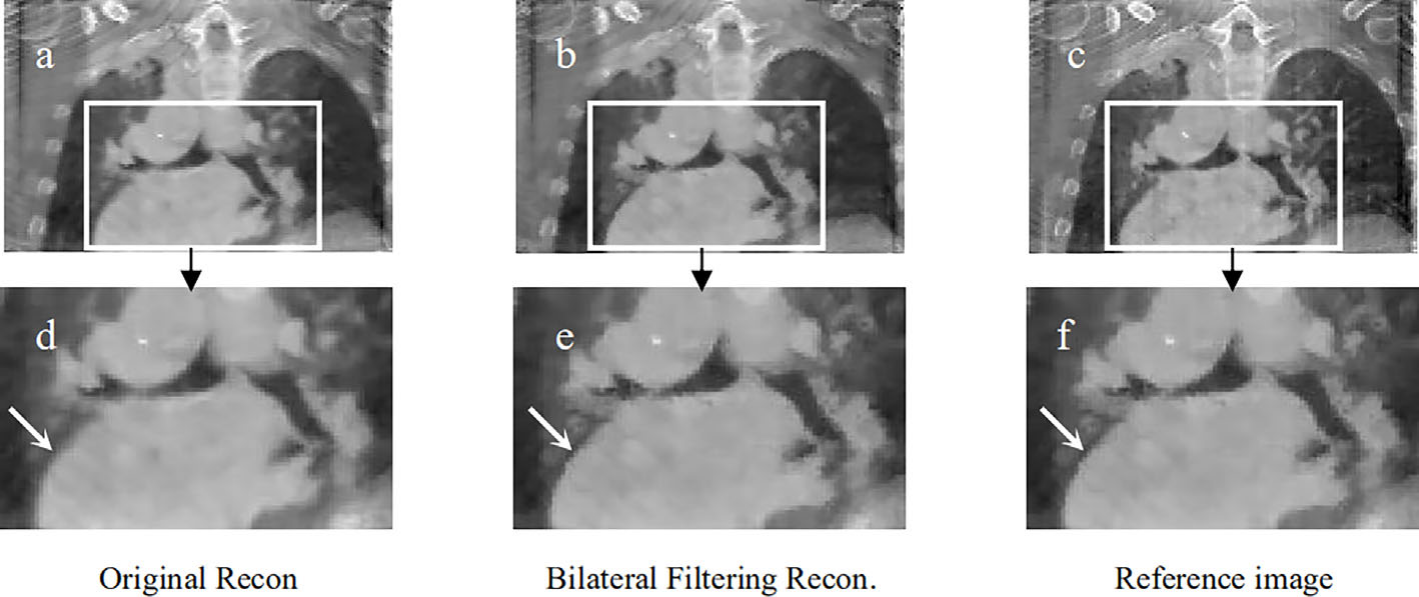
Patient 2 reconstruction comparison. **(A)** Original simultaneous reconstruction; **(B)** reconstruction with bilateral filtering; **(C)** patient reference; **(D)** resized ROI of **(A)**; **(E)** resized ROI of **(B)**; **(F)** resized ROI of **(C)**.

**Figure 8:**
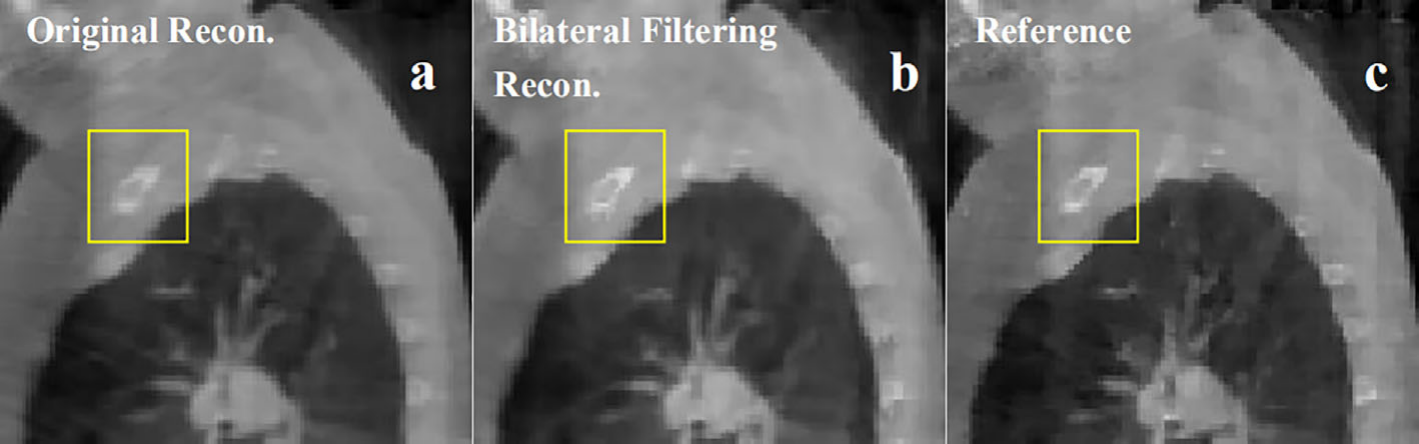
Patient 3 40% phase reconstruction results comparison. **(A)** the original simultaneous reconstruction; **(B)** the bilateral filtering based reconstruction; **(C)** patient reference.

**Figure 9:**
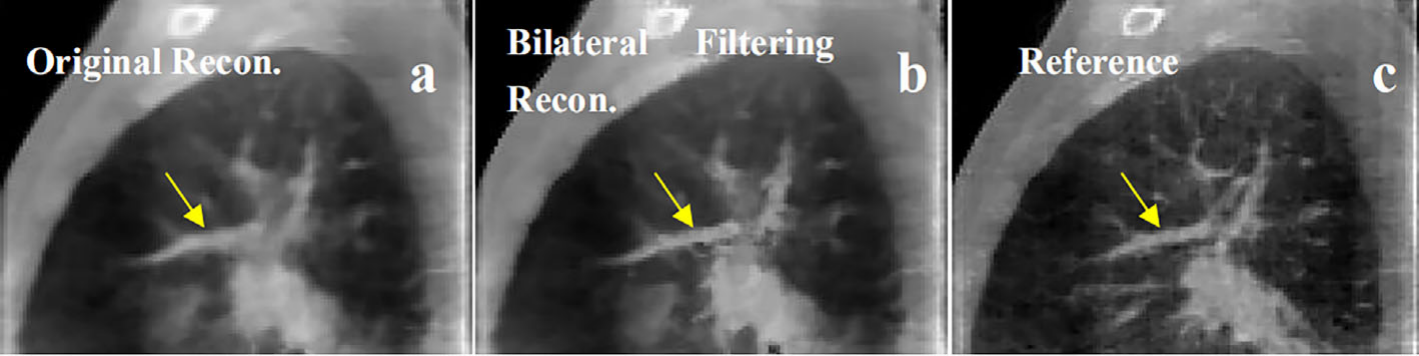
Patient 4 40% phase reconstruction results comparison. **(A)** the original simultaneous reconstruction; **(B)** the bilateral filtering based reconstruction; **(C)** patient reference.

Corresponding patient results are shown in **Figures 6****–****9**.

**Figure 6** shows the sagittal view of the reconstruction comparison of the 1st patient. **Figure 6A** shows the original reconstructed result; **Figure 6B** shows the bilateral filtering based result; **Figure 6C** shows the reference image reconstructed by TV reconstruction (3) using the fully sampled projections. The white arrow shows a tumor closely attached to the thoracic wall, and a small cavity exists between the tumor and the wall. The yellow arrow shows a side effect of bilateral filtering. And it will be discussed later in the discussion part. The corresponding reconstruction slice via TV and FDK are also listed in **Figures 6D, E**, respectively.

**Figure 7** shows the reconstructed coronal view results for the 2^nd^ patient. **Figure 7A** depicts the original simultaneous reconstructed image. **Figure 7B** shows the bilateral filtering reconstructed results. **Figure 7C** provides the patient reference. **Figures 7D–F** displayed the zoomed ROIs [refer to the ROI box in **Figure 7A**] from **Figures 7A–C**, respectively.

**Figure 8** shows the 3^rd^ patient reconstruction results. This patient case doesn't have visible sliding motion because the tumor located at the apex of lung. Hence the imaging ROI(Region of Interest) cannot observe visible sliding motion. The box ROI shows a bone structure comparison.

**Figure 9** shows the 4^th^ patient reconstruction results. The arrows labeled a fibrous structure comparison.

We summarized each patient's ROI based RE values for FDK, TV, the original simultaneous reconstruction, and the bilateral filtering based reconstruction methods in **Table 2**.

**Table 2:** RE comparison for patient data results.

RE	FDK	TV	Original Recon.	Bilateral Filtering Recon
Patient 1	29.63%	9.88%	7.05%	6.61%
Patient 2	30.55%	9.01%	7.68%	7.06%
Patient 3	30.45%	8.85%	7.21%	7.10 %
Patient 4	29.45%	9.24%	7.62%	6.98%

## Discussion and Conclusions

### Results Discussion on the Clinical Results

In **Figure 6**, compared with reference **Figure 6C** the arrow labeled small cavity in **Figure 6A** has been blurred more dramatically than that in **Figure 6B**. Meantime the image content structures in **Figure 6D** have been over-smoothed by TV reconstruction; and in **Figure 6E** all the image suffered from serious noise contamination by FDK reconstruction. The quantitative comparison in **Table 2** indicate that bilateral filtering achieve the minimum RE value, which further confirms the above subjective description of the image.

In **Figure 7**, the lung-to-heart boundary (indicated by the arrow) in **Figure 7E** is more visible than that in **Figure 7D** compared with reference **Figure 7F**. And the quantitative comparison result in **Table 2** also indicates the same trend.

The patient in **Figure 8** is a special case. The tumor in this patient is very close to the apex of the lung. So the imaging region is set to the apex region. However sliding motion can hardly be seen in the region. And we didn't find any motion difference between **Figures 8A, B**. But as bilateral filtering is capable to smooth the image while keeping sharp edges, we found in the box region the bone structures have been better reconstructed with bilateral filtering with a sharper edge (e.g. see **Figure 8B**).

In **Figure 9**, we found the fibrous structure (labeled by the arrow) has been reconstructed clearer and sharper with bilateral filtering (e.g. **Figure 9B**) than that from the original reconstruction (e.g. **Figure 9A**) compared with the patient reference (e.g. **Figure 9C**).

### σ_v_ Sensitivity Analysis for Fibrous Texture Over-Smoothing

**Figure 10:**
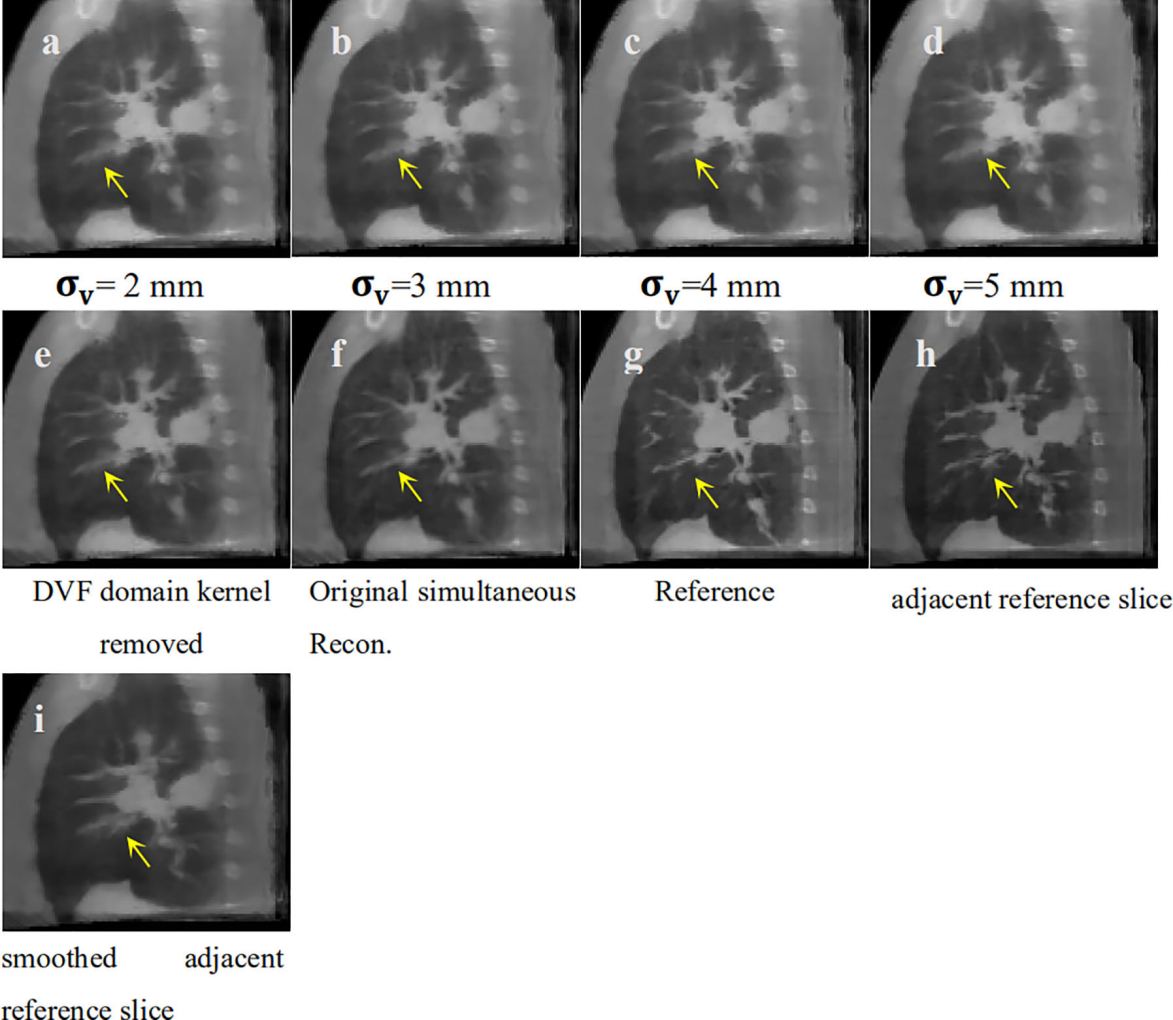
σ_v_ sensitivity analysis for texture smoothing. **(A)** Bilateral filtering based reconstruction 543 with to 2 mm; (B) Bilateral filtering based reconstruction with to 3 mm; **(C)** Bilateral 544 filtering based reconstruction with to 4 mm; **(D)** Bilateral filtering based reconstruction with 545 to 5 mm; **(E)** Bilateral filtering based reconstruction without DVF domain kernel; **(F)** Original 546 simultaneous reconstruction; **(G)** Patient Reference; **(H)** Adjacent reference slice from patient 547 reference; **(I)** Adjacent reference slice that has also been smoothed.

We discovered an over-smoothing side effect for the fibrous texture in **Figure 6**, which is labeled by the yellow arrow. We made a **σ_v_** sensitivity analysis to check whether this effect is caused by the DVF domain's filter kernel. We set **σ_v_** to 2, 3, 4, and 5 mm to perform the 4D-CBCT reconstruction. We also removed the DVF domain sub-kernel (e.g., to set the DVF domain kernel to 1) from the entire bilateral filtering kernel to perform the reconstruction and eliminate the influence of **σ_v_**. The corresponding reconstructed slices of the target phases are shown in **Figure 10**. The results indicate that regardless of how **σ_v_** changes, the arrow-labeled fibrous structure is always over-smoothed. Hence, this over-smoothing effect is not directly caused by the DVF domain sub-filter kernel. This indicates that the over-smoothing can be caused by the conventional bilateral filter's texture smoothing feature. The bilateral filtering kernel that we employed is a 3D kernel in a cubic 3x3x3 voxel region. We checked the smoothed texture's adjacent slice region and found that the adjacent local region contains dense tiny fibrous textures (refer to **Figure 10H**). The 3D bilateral filter smoothed the texture not only in the adjacent slice (**Figure 10I**) but also spread it to the current slice (**Figures 10A–D**). This over-smoothing effect occurred where the tiny fibrous textures are located very close to each other. If we want to remove this excessive smoothing effect, one possible solution is to rely on more projections within this phase. More projections will supply additional information for better reconstruction. We can also increase the image resolution by using a smaller voxel size for reconstruction.

### Reconstruction Results Comparison Between Bilateral Filtering-Based Scheme vs. Lung Segmentation-Based Scheme

**Figure 11:**
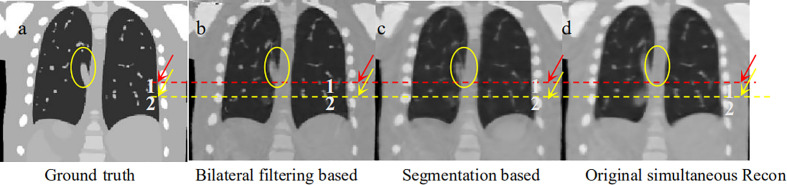
Coronal view comparison between bilateral filtering based reconstruction vs. segmentation based reconstruction. **(A)** the phantom ground truth; **(B)** the bilateral filtering based result; **(C)** the segmentation based result; **(D)** the original reconstruction result.

To make a parallel performance comparison between bilateral filtering-based reconstruction and segmentation-based reconstruction, we performed an NCAT phantom experiment. The relative reconstruction error of the bilateral filtering reconstruction is 7.3% and 7.4% for the segmentation based reconstruction. However, differences in some image slices remain. In **Figure 2** we already show that the rib can be better reconstructed with bilateral filtering reconstruction than that of segmentation result. **Figure 11** also shows the coronal views obtained from bilateral filtering-based construction, segmentation-based reconstruction, and original simultaneous reconstructions. Both of these two algorithms have corrected the rib positions to match with the ground truth (rib #1 and #2's top edges). The vein length (represented by the yellow circles) has also been corrected by these two algorithms compared with the ground truth. The vein has even been reconstructed more clearly by the bilateral filtering reconstruction. The bilateral filtering-based scheme obtained better reconstruction results compared with the segmentation based reconstruction.

### Reconstructed Image Super-Positioned With the Solved DVFs

**Figure 12:**
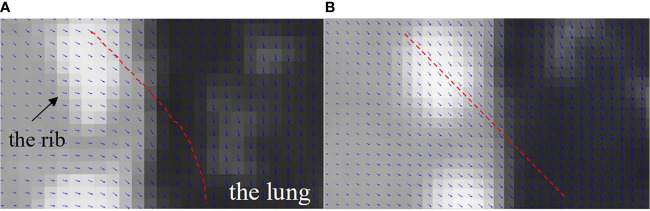
DVF super-positioned with the reconstructed images for **(A)** the bilateral filtering based reconstruction and **(B)** the original simultaneous reconstruction.

To illustrate the DVF difference between the bilateral filtering-based reconstruction and the original simultaneous reconstruction, we super-positioned their reconstructed images with their corresponding DVFs. As the sliding motion mainly occurs at the interface between the lung and the chest wall, we only focus on this zoomed local region of interest to determine the DVF differences. **Figure 12** shows the lung-chest wall ROI. **Figure 12A** is the ROI from the bilateral filtering-based result; **Figure 12B** is the corresponding ROI from the original reconstruction. The red dotted line gives the DVF flow trend. The bilateral filtering-based DVF flow (refer to red line in **Figure 12A**) drops downward from the rib side to the lung part. For the original case, the DVF flow slides straight from the rib side to the lung side. This DVF flow difference directly causes the rib position differences in the final reconstructed images. Hence, this finding reveals that bilateral filtering effectively corrected the rib position compared with the original reconstruction.

### Calculation Time

The convergence of the bilateral filtering reconstruction is similar to the original reconstruction where 200 total iterations in the DVF estimation are adequate to achieve good convergence. The computation time for one

DVF optimization iteration is 18 s for the presented algorithm to reconstruct an image with size of 200 x 200 x 150. Currently, DVFs for each phase were estimated sequentially and we partially implemented the algorithm on a GPU card (Geforce GTX 980, NVIDA, Santa Clara, CA). Only the forward projection for each view was parallel accelerated on GPU. To further speed up the calculation, possible strategy includes: 1) full GPU implementation and 2) running DVF estimation for different phases in a parallel fashion on multiple GPU cards. Recently a deep leaning based 4D-CBCT motion estimation algorithm (19, 20), was developed. In this paper a CNN model is constructed to predict a PCA (Principle Component Analysis) based DVF motion modeling. The PCA eigenvectors and the corresponding PCA coefficients are predicted to obtain the real time updated 4D-DVF. The training dataset is a pre-built projection based datasets with more than 1 × 10^6^ simulated projections from the patient 4D-CT. Their reported calculation time cost is around 30~40 min for network training with a Intel Core i7-5960X CPU, 32 GB memory and NIVIDIA GTX 1080 Ti GPU. The advantage of this algorithm is that it realized real-time motion tracking. But one disadvantage is that the training data (e.g. the simulated projections that contains respiration motion) comes from 4D-CT but not the on board 4D-CBCT. Hence once the patient respiration mode changes between the 4D-CT scanning stage and the 4D-CBCT scanning stage the predicted real time 4D-CBCT will not reflect the true respiration at the treatment stage. As the 4D-DVF estimation principle of this algorithm is fundamentally different compared with our method. So it is not fair to make a parallel comparison between our results and their algorithms. Obviously deep learning based real-time 4D-CBCT is very promising for supplying quality 4D-CBCT. Once the patient on-board respiration projection dataset were built, the deep learning network will possibly be able to predict accurate on-board 4D-CBCT. This will be our next step research focus.

### Limitation of the Patient Number for Statistical Testing

Another limitation of the current study is that the evaluation studies were performed on a limited number of patients. The CBCT scans with long acquisition times were performed under a previous institutional review board protocol. The limited number of study participants does not allow statistical testing. More patient data and statistical analysis are needed to further validate the clinical value of this method.

### How the Proposed Method Supports Clinical Translation in IGRT

Our proposed method does not need any hardware modification and employs the conventional 1 min clinical scanning protocol for imaging data acquisition. The algorithm offers physicians with high quality 4D-CBCT and it helps checking whether: 1) a patient’s respiration retains the same mode compared with his/her 4D-CT; and 2) if the tumor shape/motion mode changes obviously. This further helps the physician decide if it is safe to trigger on the SBRT beam for treatment.

## Conclusion

In this work, we proposed a bilateral filtering-based fully automatic sliding motion compensated 4D-CBCT reconstruction scheme. Both the digital NCAT phantom experiment and the pilot clinical validation demonstrated that this scheme is an effective simultaneous high-quality 4D-CBCT image reconstruction algorithm. The experiment also showed that the bilateral filtering-based algorithm outperforms the segmentation-based sliding motion modeling algorithm for 4D-CBCT reconstruction. The algorithm is a prospective 4D-CBCT tool for clinical translation in image-guided radiation therapy.

## Data Availability Statement

The data analyzed in this study is subject to the following licenses/restrictions: The dataset were from MD Anderson, and the author got the permission to use the data for this paper. The author has acknowledged the permission in the *Acknowledgments*. Requests to access these datasets should be directed to Prof. Tinsu Pan, tpan@mdanderson.org.

## Ethics Statement

The studies involving human participants were reviewed and approved by MD Anderson with IRB# 00-202. The patients/participants provided their written informed consent to participate in this study.

## Author Contributions

JD wrote the manuscript and developed most of the algorithm codes. TY is in charge of the patient data analysis and part of the manuscript revision before submission; WS developed part of the algorithm codes; HX discussed the algorithm details and revised the revision manuscript; XC and YS do the proofreading for revision. LL analyzed the patient study results; YL helped with allocating manuscript time from clinical duty of the authors; DC and TZ revised the manuscript. All authors contributed to the article and approved the submitted version.

## Funding

This work is supported by a grant from Varian Medical System, a grant from the Chongqing Municipal Human Resources and Social Security Bureau (cx2018147), a grant from Chongqing Natural Science Foundation (cstc2020jcyj-msxm2928), a seed grant from the First Affiliated Hospital of Chongqing Medical University (PYJJ2019-208); a Key Medical Projects of Jiangsu Commission of Health (No. ZDB2020022); a Key Project of Chongqing Yuzhong District Science and Technology (No.20190101); The National Natural Science Foudation of China (No. 61971078, 61501070); and The Science and Technology Foundation of Chongqing Education Commission (No. CQUT20181124).

## Conflict of Interest

The authors declare that the research was conducted in the absence of any commercial or financial relationships that could be construed as a potential conflict of interest.

## References

[1] Lu J Guerrero TM Munro P Jeung A Chi PC Balter P Four-dimensional cone beam CT with adaptive gantry rotation and adaptive data sampling. Med Phys (2007) 34(9):3520–9. 10.1118/1.2767145 17926955

[2] Li T Xing L Optimizing 4D cone-beam CT acquisition protocol for external beam radiotherapy. Int J Radiat Oncol Biol Phys (2007) 67(4):1211–9. 10.1016/j.ijrobp.2006.10.024 17197125

[3] Sidky EY Pan X Image reconstruction in circular cone-beam computed tomography by constrained, total-variation minimization. Phys Med Biol (2008) 53(17):4777–807. 10.1088/0031-9155/53/17/021 PMC2630711 18701771

[4] Sidky EY Pan X Reiser IS Nishikawa RM Moore RH Kopans DB Enhanced imaging of microcalcifications in digital breast tomosynthesis through improved image-reconstruction algorithms. Med Phys (2009) 36(11):4920–32. 10.1118/1.3232211 PMC2773453 19994501

[5] Han H Gao H Xing L Low-dose 4D cone-beam CT via joint spatiotemporal regularization of tensor framelet and nonlocal total variation. Phys Med Biol (2017) 62(16):6408–27. 10.1088/1361-6560/aa7733 PMC12184182 28726684

[6] Star-Lack J Sun M Oelhafen M Berkus T Pavkovich J Brehm M A modified McKinnon-Bates (MKB) algorithm for improved 4D cone-beam computed tomography (CBCT) of the lung. Med Phys (2018) 45(8):3783–99. 10.1002/mp.13034 29869784

[7] Leng S Zambelli J Tolakanahalli R Nett B Munro P Star-Lack J Streaking artifacts reduction in four-dimensional cone-beam computed tomography. Med Phys (2008) 35(10):4649–59. 10.1118/1.2977736 PMC2655146 18975711

[8] Zhang H Ouyang L Huang J Ma J Chen W Wang J Few-view cone-beam CT reconstruction with deformed prior image. Med Phys (2014) 41(12):121905. 10.1118/1.4901265 25471965

[9] Cai JF Jia X Gao H Jiang SB Shen Z Zhao H Cine cone beam CT reconstruction using low-rank matrix factorization: algorithm and a proof-of-principle study. IEEE Trans Med Imaging (2014) 33(8):1581–91. 10.1109/TMI.2014.2319055 PMC6022849 24771574

[10] Gao H Li R Lin Y Xing L 4D cone beam CT via spatiotemporal tensor framelet. Med Phys (2012) 39(11):6943–6. 10.1118/1.4762288 PMC3494730 23127087

[11] Dang J Gu X Pan T Wang J A pilot evaluation of a 4-dimensional cone-beam computed tomographic scheme based on simultaneous motion estimation and image reconstruction. Int J Radiat Oncol Biol Phys (2015) 91(2):410–8. 10.1016/j.ijrobp.2014.10.029 25636763

[12] Dang J Yin FF You T Dai C Chen D Wang J Simultaneous 4D-CBCT reconstruction with sliding motion constraint. Med Phys (2016) 43(10):5453. 10.1118/1.4959998 27782722 PMC5035309

[13] Wang J Gu X Simultaneous motion estimation and image reconstruction (SMEIR) for 4D cone-beam CT. Med Phys (2013) 40(10):101912. 10.1118/1.4821099 24089914

[14] Wang J Gu X High-quality four-dimensional cone-beam CT by deforming prior images. Phys Med Biol (2013) 58(2):231–46. 10.1088/0031-9155/58/2/231 23257113

[15] Papiez BW Heinrich MP Fehrenbach J Risser L Schnabel JA An implicit sliding-motion preserving regularisation via bilateral filtering for deformable image registration. Med Image Anal (2014) 18(8):1299–311. 10.1016/j.media.2014.05.005 24968741

[16] Han G LZ You J A fast ray-tracing technique for TCT and ECT studies. IEEE Nucl Sci Symp Conf Rec (1999) 3:1515–8. 10.1109/nssmic.1999.842846

[17] Emil Y Sidky XP Image reconstruction in circular cone-beam computed tomography by constrained, total-variation minimization. Phys Med Biol (2008) 53(17):4777–807. 10.1088/0031-9155/53/17/021 PMC2630711 18701771

[18] Xuejun Gu HP Liang Y Richard Castillo, Deshan Yang, Dongju Choi, Edward Castillo, Amitava Majumdar, Thomas Guerrero and Steve B Jiang. Implementation and evaluation of various demons deformable image registration algorithms on a GPU. Phys Med Biol (2010) 55(1):12. 10.1088/0031-9155/55/1/012 PMC7540904 20009197

[19] Wei R Zhou F Liu B Bai X Fu D Li Y Convolutional Neural Network (CNN) Based Three Dimentional Tumor Localization Using Single X-Ray Projection. IEEE Access (2019) 7:37026–38. 10.1109/ACCESS.2019.2899385

[20] Wei R Zhou F Liu B Bai X Fu D Liang B Real-time tumor localization with single x-ray projection at arbitrary gantry angles using a convolutional neural network (CNN). Phys Med Biol (2020) 65(6):065012. 10.1088/1361-6560/ab66e4 31896093

